# Value of trans-oesophageal echocardiography as a method of encouraging patients with chronic atrial fibrillation to use anticoagulation therapy

**DOI:** 10.5830/CVJA-2010-017

**Published:** 2010-08

**Authors:** Aurora Bakalli, Lulzim Kamberi, Gani Dragusha, Nexhmi Zeqiri, Fitim Gashi, Lazer Prekpalaj

**Affiliations:** Department of Cardiology, University Clinical Centre of Kosova, Prishtine, Kosova; Department of Cardiology, University Clinical Centre of Kosova, Prishtine, Kosova; Department of Cardiology, University Clinical Centre of Kosova, Prishtine, Kosova; Department of Cardiology, University Clinical Centre of Kosova, Prishtine, Kosova; Department of Cardiology, University Clinical Centre of Kosova, Prishtine, Kosova; Department of Cardiology, University Clinical Centre of Kosova, Prishtine, Kosova

**Keywords:** atrial fibrillation, trans-oesophageal echocardiography, left atrial appendage thrombus

## Abstract

**Background:**

Despite the indisputable role of anticoagulation therapy for atrial fibrillation (AF) patients at risk for stroke, anticoagulants remain under-used in everyday clinical practice. We assumed that by performing trans-oesophageal echocardiography (TEE) on patients with AF who were not on anticoagulation treatment prior to the procedure, and by explaining to them the TEE images obtained, as well as the possible consequences of these findings, we could convince patients to start anticoagulation therapy. The main objective of the study was to assess the examined patients’ adherence to warfarin therapy over a two-year period.

**Methods and results:**

We conducted a prospective TEE study from February 2006 to December 2008 on 70 patients with chronic AF who were not on anticoagulation treatment. Mean patient age was 65.85 ± 10.02 years and 68.57% were women. Thrombus in the left atrial appendage was found in 25 (35.71%) patients. Fifty-four (77.14%) patients had thrombi or spontaneous echo contrast in at least one of their supraventricular cavities.

Following the procedure and with detailed explanation to the patients of their TEE findings, we managed to start anticoagulation therapy on 60 (85.71%) patients. At the end of the follow-up period of 23.76 ± 2.8 months, 53 (75.71%) patients remained on warfarin therapy. The rest of the surviving patients settled for thrombo-prophylaxis with aspirin.

**Conclusion:**

TEE is a valuable method that, in addition to its diagnostic possibilities, could also serve as a convincing visual method of putting atrial fibrillation patients onto an anticoagulation regimen.

## Summary

The presence of non-valvular atrial fibrillation increases the risk of thrombo-embolism fivefold.^1^ Warfarin has been shown to be highly beneficial in the prevention of stroke or systemic thrombo-embolism in moderate- to high-risk patients with chronic atrial fibrillation. A recent meta-analysis of 29 trials, totalling 28 044 participants, which analysed the role of thrombo-prophylaxis in atrial fibrillation, showed that adjusted-dose warfarin significantly reduced the risk of thrombo-embolic events.^2^

Despite the unquestionable role of anticoagulation therapy in patients with non-valvular atrial fibrillation at moderate to high risk of stroke, it still remains under-used in clinical practice.^3^,^4^ Studies on clinical practice report that only a quarter to a half of eligible patients with atrial fibrillation undergo anticoagulation treatment.^5^,^6^ Under-usage of anticoagulation therapy can be attributed to two main aspects: physician and patient reluctance.

The aim of this study was to perform trans-oesophageal echocardiography (TEE) in patients with chronic non-valvular atrial fibrillation who did not take anticoagulation therapy prior to the procedure, in order to asses the prevalence of atrial thrombi and spontaneous echo contrast (SEC) in these patients. The main purpose was to evaluate their adherence to anticoagulation therapy after TEE, as well as to assess the morbidity and mortality associated with atrial fibrillation over a two-year follow-up period following the TEE procedure. Our hypothesis was that detection of thrombi or SEC by TEE would serve as a persuasive tool to convince the patient of the importance of anticoagulation therapy.

## Methods

We conducted a prospective TEE study from February 2006 to December 2008 on 70 patients with chronic non-valvular atrial fibrillation at moderate to high risk of stroke, who were not on anticoagulation therapy. TEE procedures were carried out from February to October 2006; thereafter these patients regularly visited an outpatient cardiology ward for a mean period of two years. The emphasis was on assessment of their adhesion to anti-thrombotic therapy.

Atrial fibrillation was diagnosed by at least two electrocardiograms (ECG), presenting with the absence of P waves before each QRS complex, and replaced instead by fibrillatory ‘f ’ waves varying in size, shape and timing. Patients at moderate to high risk of stroke were those who, in addition to chronic non-valvular atrial fibrillation, also suffered from arterial hypertension, diabetes mellitus, vascular disease, heart failure, or previous stroke, transient ischaemic attack or systemic thrombo-embolic event. Patient age was also taken into consideration when stratifying them into moderate or high stroke-risk category.

Comprehensive histories, physical examinations, laboratory tests, electrocardiograms, transthoracic echocardiography (TTE) and TEE were performed on all patients included in the study. Patients who could not tolerate TEE were excluded. Written consent was obtained from all patients in the study.

Patients were asked specifically if they were aware of the importance of antithrombotic therapy for their medical condition, if their managing physician had suggested they start on anticoagulation therapy, and if this was so, if the patient had declined anticoagulant treatment.

## Echocardiographic studies

Conventional TTE and TEE were carried out in the presence of two skilled cardiologists using the Philips ie33 system. The TTE measurements were obtained from the parasternal long-axis view by two-dimensional targeted M-mode tracing according to the recommendations of the American Society of Echocardiography. TEE was performed using a multiplane probe with a 7.0-MHz transducer.

All patients had fasted on the day of the TEE procedure. They were put under conscious sedation by intravenous Midazolam injection, which was given in a range from 1.5–5 mg. Topical anesthesia of the hypopharynx was achieved by lidocaine spray.

The left atrial appendage (LAA), as the major location of cardiac thrombi in patients with chronic non-valvular atrial fibrillation, was visualised from the two-chamber longitudinal view of the left atrium and left ventricle. Thrombus was defined as the presence of a distinct, well-contoured echogenic mass, identified in at least two different views. The presence of spontaneous echo contrast (SEC) was described as dynamic ‘smoke-like’ echoes with swirling motion in the cavity. The impact of the white-noise artifact was excluded by adjusting the gain setting as required.

After the procedure, all acquired trans-oesophageal images were carefully explained to the patient and a companion. After elaborating on the possible consequences of the pathologies that were detected, patients were advised to start on anticoagulation treatment.

## Statistical analysis

All values were expressed as means ± SD or fractions. Statistical analyses were performed using statistical software (SSP, version 2.80, 2005).

## Results

Seventy patients with chronic atrial fibrillation took part in this study. Mean patient age was 65.85 ± 10.02 years, and 68.57% were women. Other baseline patient characteristics are shown in Table 1. None of the patients was taking warfarin prior to the study; 61 (87.14%) patients were on aspirin before entering the study.

**Table 1.** Baseline Patient Characteristics

	n = 70
Age (years)	65.85 ± 10.02
Female (%)	48/70 (68.57)
Male (%)	22/70 (31.43)
Hypertension (%)	54/70 (77.14)
Coronary artery disease (%)	29/70 (41.43)
Diabetes mellitus (%)	25/70 (35.71)
Heart failure (%)	20/70 (28.57)
History of stroke (%)	9/70 (12.86)

Data are presented as mean ± SD or *n* (%).

Forty-eight (68.57%) patients were not aware of the importance of anticoagulation therapy for their condition. Thirty-six (51.43%) patients admitted that were advised by their physician to start on anticoagulation therapy. Patient refusal to take warfarin was mostly because of the fear of haemorrhage or the inconvenience of INR monitoring, due to frequency of monitoring or distance of the INR monitoring centre from the patient’s residence.

Mean left atrial diameter of the included patients was 51.74 ± 7.46 mm. SEC was present most frequently in the left atrium (in 64.29% of patients). Fifty-two (74.29%) patients had SEC in the left or right atrium, whereas 33 (47.14%) had SEC in both atria. Thrombi were found most frequently in the left atrial appendage (25 cases) (Table 2), whereas 32 (45.71%) patients had thrombi in either the left or right atrial appendage. Additional transoesophageal data are presented in Table 2. Fifty-four (77.14%) patients had thrombi or SEC in at least one of their supraventricular cavities.

**Table 2.** Trans-Oesophageal Data Of The Patient Population

	n = 70
LAD, TEE (mm)	51.74 ± 7.46
LAA maximal area (cm^2^)	4.3 ± 1.93
RAA maximal area (cm^2^)	0.99 ± 0.6
LA SEC (%)	45/70 (64.29)
RA SEC (%)	40/70 (57.14)
LA or RA SEC (%)	52/70 (74.29)
LA and RA SEC (%)	33/70 (47.14)
LAA thrombus (%)	25/70 (35.71)
RAA thrombus (%)	15/70 (21.43)
LAA or RAA thrombus (%)	32/70 (45.71)
LAA and RAA thrombus (%)	8/70 (11.43)

Data are presented as mean ± SD or *n* (%).

The mean follow-up period was 23.76 ± 2.8 months. During this period, one patient died as a consequence of ischaemic stroke. TEE was performed one month prior to the event and the deceased 66-year-old female was diagnosed with LAA thrombus, as well as the presence of SEC in both atria. One patient suffered an ischaemic stroke and two patients experienced transient ischaemic attacks over the follow-up period. Two patients were hospitalised due to signs and symptoms of heart failure.

Following the procedure and after thorough explanation of the TEE findings to the patients, we managed to start anticoagulation therapy on 60 (85.71%) patients. Thirty-five (50%), in addition to warfarin, took low-dose aspirin. The 10 (14.29%) patients who did not agree to start on anticoagulation with warfarin settled for thrombo-prophylaxis with aspirin.

At the end of the follow-up period, 53 (75.71%) patients remained on warfarin therapy. Five had stopped the anticoagulation regimen due to minor bleeding problems, while two patients had preferred to stop warfarin therapy due to an inability to have regular INR monitoring. However, these patients agreed to take low-dose aspirin.

## Discussion

Non-valvular atrial fibrillation increases the risk of stroke four- to fivefold in all age groups,^1^,^7^ with the main source of thrombi being cardio-embolic, originating mostly from the left atrial appendage.^8^ Despite abundant information from ongoing clinical trials in favour of warfarin use in patients with chronic atrial fibrillation to prevent stroke, it remains a concern how to approach patients in order to implement the anticoagulation strategy in everyday clinical practice. The prescription rate of anticoagulation therapy for eligible atrial fibrillation patients is only 15 to 44%.^9^

Under-utilisation of anticoagulation therapy is due to either physician or patient reluctance. Factors contributing to physician under-prescription rates of warfarin therapy are mostly overestimation of the associated risks of warfarin therapy,^10^ or underestimation of the stroke risk.^11^

An important factor in patient reluctance, contributing to under-usage of anticoagulation therapy for chronic atrial fibrillation, was the lack awareness of thrombo-prophylaxis for this condition.^12^ Studies have shown that very few elderly patients with chronic atrial fibrillation realised the risk–benefit ratio of anticoagulation treatment.^13^ These data coincide with our findings, where 68.57% of patients with atrial fibrillation at moderate to high risk of stroke had no information concerning the importance of anticoagulation therapy. Even if the patient is well informed by the physician about the benefits of warfarin for their condition, they may still refuse to take the medication due to the difficulties of INR monitoring or adjustment to the dietary lifestyle.^14^

Pictograms have been shown to be a useful way of representing the risk–benefit ratio of warfarin for atrial fibrillation, thereby making the importance of anticoagulation treatment more understandable to patients.^13^ However, studies have demonstrated that providing additional information on warfarin use, such as the need for regular INR monitoring or abstinence from alcohol had a negative effect on the patient’s decision to opt for anticoagulation therapy.^15^

The large proportion of uneducated older people in Kosova, particularly women, presents a problem for physicians prescribing anticoagulation therapy for patients with chronic atrial fibrillation. This makes it challenging to explain to their patients the risk–benefit ratio of warfarin usage in numerical terms. Therefore, we hypothesised that by creating visual images of their condition, patients would agree to start on anticoagulation treatment.

After the TEE procedure, the images were carefully explained to the patient and an accompanying person. The presence of thrombus was described as a potential cause of ischaemic stroke or other thrombo-embolic event, SEC was explained as a precursor of thrombus, and enlargement of the atria or atrial appendages was represented as a favourable condition for future thrombus formation.

This method proved to be effective in achieving a 75.71% rate of adherence to warfarin therapy and 100% to thromboprophylaxis with either warfarin or aspirin in our study population over a two-year follow-up period. We believe that our results demonstrate the importance of routine clinical use of TEE in patients with chronic non-valvular atrial fibrillation, as a tool to convince these patients of the need for anticoagulation treatment.

One limitation of this study was that a control group was not formed. Another was that a relatively small number of patients were included in the study.

## Conclusion

TEE is a valuable method not only to asses the presence of thrombi in the atrial appendage or SEC in patients with chronic atrial fibrillation, but is also a handy visual means of persuading patients to commence anticoagulation treatment.
